# Black Tea and Theaflavins Assist Healing of Indomethacin-Induced Gastric Ulceration in Mice by Antioxidative Action

**DOI:** 10.1155/2011/546560

**Published:** 2010-09-29

**Authors:** Biplab Adhikary, Sudhir Kumar Yadav, Kshama Roy, Sandip K. Bandyopadhyay, Subrata Chattopadhyay

**Affiliations:** ^1^Department of Biochemistry, Dr. B.C. Roy Post Graduate Institute of Basic Medical Sciences & IPGME&R, 244B, Acharya Jagadish Chandra Bose Road, Kolkata 700 020, India; ^2^Bio-Organic Division, Bhabha Atomic Research Centre, Mumbai 400 085, India

## Abstract

The healing activities of black tea (BT) and the theaflavins (TF) against the indomethacin-induced stomach ulceration were studied in a mouse model. Indomethacin (18 mg/kg, p.o.) administration induced maximum ulceration in the glandular portion of the gastric mucosa on the 3rd day, accompanied by increased lipid peroxidation and protein oxidation, depletion of thiol-defense and mucin, as well as reduced expressions of cyclooxygenases (COX) and prostaglandin (PG) E synthesis in the gastric tissues, and plasma total antioxidant status of mice. Treatment with BT (40 mg/kg), TF (1 mg/kg), and omeprazole (3 mg/kg) produced similar (74%–76%) ulcer healing, as revealed from the histopathological studies. Treatment with all the above samples reversed the adverse oxidative effects of indomethacin significantly. BT and TF also enhanced the PGE synthesis by augmenting the expressions of COX 1 and 2, but did not modulate acid secretion.

## 1. Introduction

Stomach ulceration induced by nonsteroidal anti-inflammatory drugs (NSAIDs) is a major medical problem, ranking fourth in terms of causing morbidity and mortality [1]. The NSAID-related gastroduodenal damage is very frequent, and the most serious complication of any drug therapy. The NSAIDs mainly cause upper gastrointestinal (GI) complications, ranging from dyspeptic symptoms in up to 40%, to peptic ulceration in 20–30% of the chronic NSAID users, and even duodenal ulcers. Currently, the use of NSAIDs accounts for approximately 25% of gastric ulcer cases [2, 3]. The commercially available synthetic antiulcer drugs show side effects [4] and cannot prevent ulcer recurrence. Hence, there is a need to develop alternative herbal formulations. For centuries traditional medical systems are being used to treat various diseases in the countries of their origin. Despite the present dominance of the scientifically proven therapies, there is a renewed public interest in complementary and alternative medicines even in the Western world [5, 6]. This has been prompted, in part, by increased side effects and high cost of new drugs, lack of curative treatment for several chronic diseases, microbial resistance, and emerging diseases.

Several plants and herbs are used in traditional medicine to treat GI disorders. Documented scientific studies have demonstrated that many of these medicinal plants and their constituent phytoceuticals not only provide gastroprotection against various ulcerogens, but also accelerate ulcer healing [7, 8]. Many taxa of medicinal plants have been assessed worldwide for their antiulcerogenic effects [9, 10]. The traditional Japanese medicine, Rikkunshito, is used in Japan to treat various GI disorders, while the unripe fruits of *Carica papaya*, extensively used in India for stomach problems, has been suggested to be beneficial in gastric ulcer [11, 12]. Besides the use of NSAIDs, *Helicobacter pylori* infection is perhaps the most significant cause for gastric ulceration, leading to even gastric cancer. Different alternative and traditional medicines are being critically analyzed for their efficacy *vis-à-vis *the currently used triple therapy to eradicate the bacteria [13, 14].

For decades, doctors have recommended dietary adjustments aimed at preventing or treating symptoms of gastritis and ulceration, as diet may moderate the risk for gastritis or peptic ulcer [15]. *Camellia sinenesis* is widely grown in the tropical humid climate of South East Asia, and decoction of its leaves (tea) is the most popular nonalcoholic beverage worldwide. Tea is consumed in three basic forms: green tea, black tea, and oolong tea. Extensive work has been carried out regarding various medicinal attributes of green tea. Of these, its protective capacity against cancer [16] and cardiovascular disease [17] are of contemporary significance. The cytoprotective action of the green tea catechins against ethanol- or restraint plus water-immersion stress-induced acute gastric mucosal injury and acetic acid-induced chronic gastric ulcers in rats has been reported [18]. In addition, epigallocatechin gallate, a constituent of green tea, has been suggested to control *H. pylori*-related chronic inflammations or regress cancer precursor lesions, while a pectin-type acidic polysaccharide from green tea is reported to posses antiadhesive effects against *H. pylori *[19]. It is believed that polyphenols or polyphenol derivatives from green tea may be useful in either the prevention or the treatment of *H. pylori*-associated gastric diseases. Many of the health benefits of green tea are attributed to its antioxidant property [20]. Although black tea (BT) accounts for 80% of the total tea consumption, studies on the pharmacological properties of BT are scarce. The prophylactic action of the tea seed-derived triterpene saponins against ethanol-induced gastric mucosal lesions [21, 22] and of BT extract against various ulcerogens [23, 24] have been reported in rat models. The Food and Agricultural Organisation (FAO) of the United Nations has stressed the need for research on the health benefits of BT in its totality, and not on certain isolated fractions/constituents [25]. In the recent years there has been a mounting interest in exploring the possibility of using BT as a supplement among patients. 

The tea polyphenols, especially the catechins are primarily responsible for the curing property of green tea [26]. However, during the production of BT, a significant part of the catechins is converted to the theaflavins (comprising of theaflavin-3-gallate, theaflavin-3′-gallate, and theaflavin-3,3′-digallate) and thearubigins by a polyphenol oxidase [27]. Hence, one of the aims of the present study was to evaluate the healing property of BT and its major constituent, the theaflavins (TF) *vis-à-vis* that of omeprazole (Omez) against indomethacin-induced acute gastric ulceration in mice. Given the complexity of the ulcer healing process, the reported [24] cytoprotective property of BT against stomach ulceration does not guarantee its healing potency. Factors such as oxidative stress and reduced PG synthesis contribute to the NSAID-induced gastropathy. Hence the role of BT and TF in modulating these biochemical parameters was also investigated. Our results revealed that the biochemical transformation of green tea into BT did not hamper the ulcer healing property, and also established TF as the active antiulcerogenic principle of BT. Both BT and TF were found to exert their action by augmenting mucosal antioxidant defense, protecting mucin and increasing the cyclooxygenase (COX-) derived prostaglandin E (PGE) synthesis.

## 2. Methods

### 2.1. Chemicals and Reagents

Leaves of *C. sinenesis* (Brooke Bond, Red label) procured from the local market were identified by hptlc and hplc analyses of its chemical constituents. Alcian blue, indomethacin, bovine serum albumin (BSA), haematoxylene, alum, eosin, butylated hydroxytoluene (BHT), guanidine hydrochloride, trifluoroacetic acid (TFA), omeprazole (Omez), TF, and sucrose were procured from Sigma, St. Louis, MO. Other reagents used were 2-thiobarbituric acid (TBA), ethanol, butanol and ethyl acetate (E. Merck, Mumbai, India), trichloroacetic acid (TCA, Thomas Baker, Mumbai, India), hydrogen peroxide (35%, Lancaster, Morecambe, UK), 2,4-dinitrophenyl hydrazine (DNPH), disodium hydrogen phosphate and sodium dihydrogen phosphate (BDH, Mumbai, India), antibodies for COX-1 and COX-2 (Santa Cruz Biotechnology, Santacruz, CA), Lumi-Light^PLUS^ western blotting kit (Roche Applied Science, Baden-Wurttemberg, Mannheim), nitrocellulose membrane (BioTraceNT) (Pall Life Sciences, Easthills, NY), *β*-actin antibody (Cell Signaling Technology Inc., Danvers, MA), and PGE metabolite EIA kit (Cayman Chemical, Ann Arbor, MI).

### 2.2. Preparation of BT Extract

Tea leaves (30 g) were soaked in preheated (102°C) distilled water (100 mL), allowed to stand for 5 min, and the supernatant decanted. The process was repeated two times and the combined aqueous extracts were lyophilized to get BT as a sticky dark brown solid.

### 2.3. Chemical Composition of BT

#### 2.3.1. Total Phenolics and Flavonoids Contents (TPC and TFC)

Following a known method [28] the amounts of total phenolics in BT was determined. Gallic acid monohydrate was used as the standard and the TPC is expressed as mg gallic acid equivalent (GAE)/g of BT.

The known method [29] downscaled to 1 mL was followed to estimate TFC. To a solution of BT (100 *μ*g) in 0.4 mL distilled water at 25°C was added NaNO_2_ (0.03 mL, 5%), followed by AlCl_3_ · 6H_2_O (0.03 mL, 10%), after 5 min. After incubating for 6 min, aqueous NaOH (0.2 mL, 1 M) was added, and the mixture was diluted to 1 mL with water. The TFC was estimated from the absorbance of the mixture at 510 nm, using epicatechin as the standard. The TFC value is expressed as mg epicatechin equivalent (ECE)/g of BT.

#### 2.3.2. Chromatographic Characterization

BT (0.372 g) was successively extracted with ethyl acetate and butanol (each 5 mL, 3 times). The individual extracts were concentrated in vacuo, and the butanol-soluble fraction was analyzed by HPTLC (Camag Instrument, RP-18 silica gel G plate, ethyl acetate : methanol : water  =  10 : 1.1 : 1 as the solvent). The major chemical constituents were identified by comparing the retention times of the commercially available authentic compounds, under identical conditions. For quantification, standard graphs of the authentic samples were used.

### 2.4. Ulcer Healing Activity

#### 2.4.1. Test Samples Preparation

For the ulcer-healing experiments, the test samples (BT, TF, and Omez) were prepared as aqueous suspensions in 2% gum acacia as the vehicle, and administered to the mice orally.

#### 2.4.2. Animals

Male Swiss albino mice, bred at BARC Laboratory Animal House Facility, Mumbai, India, were procured after obtaining clearance from the BARC Animal Ethics Committee (BAEC). All the experiments were conducted with strict adherence to the ethical guidelines laid down by European Convention for the Protection of Vertebrate Animals used for Experimental and Other Scientific Purposes. In addition, the ethical guidelines, laid down by the Committee for the Purpose of Control and Supervision of Experiments on Animals (CPCSEA), constituted by the Animal Welfare Division, Government of India, on the use of animals in scientific research were followed. The mice (6–8 weeks old, 25–30 g) were reared on a balanced laboratory diet as per National Institute of Nutrition, Hyderabad, India, and given tap water ad libitum. They were kept at 20 ± 2°C, 65%–70% humidity, and 12 h day/12 h night cycles. The experiments were performed by two investigators blinded to the group and treatment of animals, which were identified by typical notches in the ear and limbs (performed at a preweaning stage to minimize the pain to the animals), and then randomized.

#### 2.4.3. Ulceration Protocol

The mice were divided into several groups (each containing five mice), and each experiment was repeated three times. Except for the normal control, ulceration in the other mice was induced by indomethacin (18 mg/kg, p. o., single dose), dissolved in distilled water and suspended in 2% gum acacia as the vehicle. For the standardization of doses, BT (10–50 mg/kg, p. o.) or TF (0.5–5.0 mg/kg, p. o.) were given to the mice once daily up to 7 days, starting the first dose 6 h after the indomethacin-administration. In the subsequent days, the test samples were given at 9 AM on each day. Omez (3.0 mg/kg, p. o.) was used as the positive control. The doses of indomethacin and Omez were standardized in our earlier study [30]. The normal and ulcerated control groups of mice were given the vehicle (0.2 mL) during the entire period of study. Four hours after the last dose of the test samples, the mice were sacrificed on the 3rd, 5th, and 7th days under anesthesia with thiopental, the stomachs were opened along the greater curvature, thoroughly rinsed with normal saline, and the wet weights of the tissues were recorded.

#### 2.4.4. Ulcer Healing Assessment

The gastric mucosal areas were visualized using a transparent sheet and a dissecting microscope. The extent of healing was assessed from the MDS of the untreated and treated mice. The gastric injury (MDS) was scored [31] by grading on a 0–4 scale, based on the severity of hyperemia and hemorrhagic erosions: 0—almost normal mucosa, 0.5—hyperemia, 1—one or two lesions, 2—severe lesions, 3—very severe lesions, 4—mucosa full of lesions (lesions—hemorrhagic erosions, hyperemia—vascular congestions). The macroscopic data are presented as mean ± S.E.M. from the review of a minimum of three sections per animal and five animals per group.

For histopathology, the ulcerated portions of the stomach were fixed in 10% formol saline solution for 24 h, embedded in paraffin blocks, and cut into 5 *μ*m sections. These were placed onto glass slides, stained with haematoxylene and eosin, and viewed under a light microscope. Histological sections were coded to eliminate an observer bias.

### 2.5. Biochemical Analyses

The MDS results revealed peak ulceration, and also maximum ulcer healing by the test samples on the 3rd day after indomethacin administration. Hence, we assessed the biochemical parameters on the 3rd day of ulceration under the optimized doses of the test samples (BT (40 mg/kg), TF (1 mg/kg), and Omez (3 mg/kg)). For this, the mice were equally divided into five groups as follows.

Group I—normal mice; Group II—ulcerated mice; Groups III-V—ulcerated mice, treated with BT, TF, and Omez, respectively. The total antioxidant status (TAS) was measured using plasma, while PGE was determined using both serum and tissue lysate. The other biochemical parameters were analyzed using the ulcerated portions of the glandular stomach tissues of the mice.

#### 2.5.1. TAS Assay

Following a reported method [32] and manufacturer's instructions, the TAS of plasma (mmol/L) was measured using a Randox kit. Briefly, plasma (20 *μ*L) or the standard (6-hydroxy-2,5,7,8-tetramethylchroman-2-carboxylic acid, 1.65 mmol/L) or reagent blank (double-deionized H_2_O) were mixed with 1 mL chromogen (metmyoglobin, 6.1 *μ*mol/L and 2,2′-azino-di[3-ethylbenzthiazoline] sulphonate, 610 *μ*mol/l). After mixing, the initial absorbance (A1) at 600 nm was read at 37°C. Hydrogen peroxide (200 *μ*L, 250 *μ*M) was added to the sample/standard/blank, and the absorbance (A2) was read exactly after 3 min. Subtraction of the respective A2 values from A1 gave the absorbance of sample/standard/blank. The respective TAS was obtained using the formulae: TAS  =  factor × (absorbance of blank − absorbance of sample) mmol/L; factor  =  concentration of standard/(absorbance of blank − absorbance of standard).

#### 2.5.2. Protein and Lipid Damages Assay

The glandular stomach tissues from five animals of each group were pooled, rinsed with phosphate buffer (50 mM, pH 7.4), homogenized in the same buffer with a glass-Teflon homogenizing tube, and centrifuged at 1200 × g to obtain the supernatant. The amount of protein carbonyls was determined using a known method [33]. Briefly, DNPH (4 mL, 10 mM) in 2 M HCl was added to the supernatant (1.0 mL), which was incubated for 1 h with intermittent shaking. Ice-cold 20% aqueous TCA solution (5 mL) was added and the mixture incubated for 15 min. The precipitated protein was washed three times with a mixture of ethanol-ethyl acetate (1 : 1), and subsequently dissolved in a solution (1 mL) containing 6 M guanidine HCl in 20 mM potassium monobasic phosphate, adjusted to pH 2.3 with TFA. After centrifuging, the amount of protein carbonyl was determined from the absorbance of the supernatant at 362 nm (*ϵ* = 2.2 × 10^4^ M^−1^ cm^−1^).

For the analysis of lipid peroxidation (measured in terms of thiobarbituric acid reactive species (TBARS)), a 10% homogenate of the glandular stomach tissues was prepared in a buffer containing (320 mM sucrose, 5 mM HEPES, 20 mM EDTA, and 0.01% BHT). The samples were centrifuged at 1200 × g for 15 min, and the supernatant centrifuged again at 12000 × g for 30 min to obtain the mitochondrial pellets. These were washed with a buffer (150 mM KCl and 20 mM phosphate buffer) and finally suspended in a phosphate buffer (50 mM, pH 7.4). The mitochondrial membrane fraction (1 mL) was treated with TCA/TBA/HCl (2 mL, 15% TCA, 0.375% TBA, 0.25N HCl) containing 0.01% BHT, heated on a boiling water bath for 15 min, cooled, and centrifuged at 3000 × g for 5 min. The amount of TBARS was calculated from the absorbance of the supernatant at 535 nm (*ϵ* = 1.56 × 10^5^ M^−1^ cm^−1^).

#### 2.5.3. Nonprotein Thiol (NP-TSH) Assay

Following a reported method [34], the gastric mucosal NP-TSH was measured. Briefly, the glandular stomach homogenates were prepared in 0.2 M Tris-HCl buffer, pH 8.2 containing 20 mM EDTA and centrifuged at 1200 × g for 15 min. An aliquot of the homogenate (1 mL) was treated with ice-cold 20% TCA (1 mL), centrifuged at 3000 × g for 5 min, and the supernatant (1 mL) was added to Tris-HCl buffer (2 mL, 0.8 M, pH 9) containing 20 mM EDTA, and mixed with DTNB (0.1 mL, 10 mM). The NP-TSH content was calculated from the absorbance of the chromogen at 412 nm (*ϵ* = 13.6 × 10^4^ M^−1^ cm^−1^).

#### 2.5.4. Mucin Assay

Following a reported method [35], the free mucin content in the gastric tissues was estimated by measuring the amount of alcian blue bound to mucus. Briefly, the glandular stomach tissues were incubated with a 1% buffered sucrose solution of alcian blue in (0.1%) sodium acetate at 37°C for 60 min. After incubation, the tissues were washed with sucrose and centrifuged. The supernatant was extracted with MgCl_2_, and the amount of alcian blue was estimated spectrophotometrically at 610 nm. The quantity (*μ*g) of alcian blue/g of wet glandular tissue was calculated.

#### 2.5.5. Western Blots

Equal amounts of glandular stomach tissue lysates (80 *μ*g) were separated by 12% SDS-polyacrylamide gel electrophoresis, and electrotransferred to nitrocellulose membrane. The membranes were blocked for 1 h at room temperature in TBST buffer (10 mM Tris-HCl, pH 8.0, 150 mM NaCl, and 0.1% Tween-20) containing 5% (w/v) nonfat milk, and incubated overnight at 4°C with appropriate primary antibodies (1 : 3000). After several washes, HRP-conjugated secondary antibody (1 : 5000) was added, the membranes were incubated further for 1 h, and the blots were developed using a Lumi-Light^PLUS^ western blotting kit. The bands were quantified with respect to that of *β*-actin bands, using a Kodak Gelquant software. The values (arbitrary unit, mean ± S.E.M.) are the density scanning results of three independent experiments, considering that of normal mice as 1.

#### 2.5.6. PGE Assay

Following harvesting of the stomach, the corpus (full thickness) was excised, weighed (~100 mg), and homogenized in 10 mM sodium phosphate buffer, pH 7.4 (1 mL). After centrifugation (9000 × g), the PGE level in the supernatant was measured by ELISA, and the concentration is expressed as pg/mg protein. The PGE level in the sera was also measured similarly and the value is expressed as pg/mL.

### 2.6. Statistical Analysis

The data are presented as mean ± S.E.M. The biochemical data were analyzed using a paired “*t*” test for the paired data or one way analysis of variance (ANOVA) followed by a Dunnet multiple comparisons post test. Nonparametric data (MDS) were analyzed using Kruskal-Wallis test (nonparametric ANOVA) followed by a Dunn's multiple comparisons post test. A probability value of *P* < 0.05 was considered significant.

## 3. Results

### 3.1. Chemical Analysis of BT

The ethyl acetate extract of BT provided a negligible amount of the residue, while the butanol extract furnished a fraction in 0.73% yield. The HPTLC analyses of this fraction revealed caffeine (Rf 0.39) as its major (70%) component along with the TFs (~18%, Rfs 0.04, 0.08, and 0.11). In addition, the fraction also contained epigallocatechin (Rf 0.18, 1.8%), catechin (Rf 0.20, 2.9%), and epicatechin (Rf 0.25, 5.5%), along with two less polar unidentified compounds (Rfs 0.47 and 0.63) in traces. The components were identified by comparison with commercially available authentic samples. This was also confirmed by the HPLC analysis. The TPC and TFC values of BT were 222.03 ± 6.31 mg GAE/g of BT and 77.95 ± 4.17 mg ECE/g of BT, respectively.

### 3.2. Both BT and TF Heal Indomethacin-Induced Gastric Ulcers in Mice

#### 3.2.1. Dose Standardization

**Figure 1:**
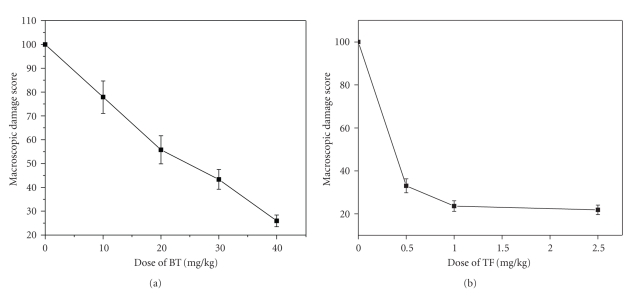
Concentration-dependent healing capacities of (a) BT and (b) TF on the 3rd day after indomethacin-induced stomach ulceration in mice. Ulceration in the mice was induced by indomethacin (18 mg/kg, *p. o*.). The healing capacity was calculated from macroscopic damage scores (MDS), measured 4 h after the last dose of the test samples. The MDS in ulcerated untreated mice was taken as 100. The values are mean ± S.E.M of three independent experiments, each with 5 mice/group. The IC_50_ values (concentration that produces 50% ulcer healing) of BT and TF (determined by Probit analysis) were significantly different (*P* < 0.01).

The doses of the test samples for effective ulcer healing were optimized by carrying out the treatment with different doses of BT (10–50 mg/kg) and TF (0.5–5.0 mg/kg) up to seven days, and the results are presented in Table 1. Omez was used as the positive control. The mice receiving only vehicle showed no mucosal lesions. Indomethacin (18 mg/kg) administration produced acute lesions in the gastric mucosa of the mice, measured in terms of MDS. Treatment with the test samples for 3 days accelerated the healing of gastric lesion dose-dependently. Overall, treatment with BT (40 mg/kg) and TF (1.0 mg/kg) for 3 days after ulcer induction provided optimal and comparable (74.1% and 76.4%, resp.) ulcer healing, which did not improve much even at their respective highest doses. The 3 day-treatment with Omez (3.0 mg/kg) produced 74.7% ulcer healing. Extending the treatment up to seven days with the respective optimized doses of BT and TF led to only marginally better healing than that observed with the three-day treatment regime. However, a major part of this was due to natural healing, with less contribution by the test samples. Hence, all subsequent experiments were carried out with the optimized dose of the test samples. Considering the MDS values of the 3rd day-ulcerated untreated mice as 100%, the IC_50_ values of BT and TF were found to be 24.5 ± 2.79 and 0.38 ± 0.05 mg/kg, respectively, (Figures 1(a) and 1(b)). 

#### 3.2.2. Histological Assessment

**Figure 2:**
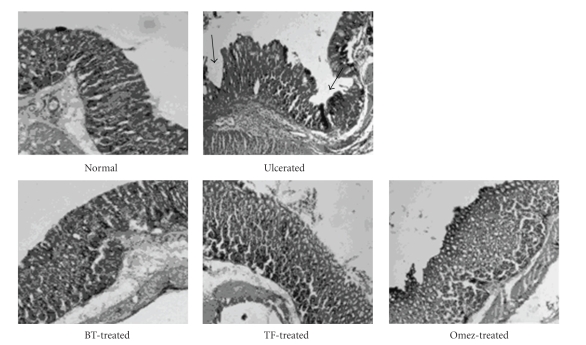
Histological assessment of acute gastric mucosal injury induced by indomethacin (18 mg/kg, *p. o*.) in mice and its prevention by BT (40 mg/kg), TF (1 mg/kg), and Omez (3.0 mg/kg). The section of mice stomachs were dissected 4 h after the last dose of the respective test samples on the 3rd day of ulceration. Black arrows indicate mucosal damage.

Within 6 h after indomethacin administration, superficial erosion and mild inflammation in the stomach were observed, indicating acute ulceration (figure not shown). However, on the 3rd day, marked damage to the glandular portion of the gastric mucosa was noticed in the histological photograph of the stomach sections of the 3rd day-ulcerated group of mice. Multiple punched-out areas of ulceration with inflammatory infiltrate containing neutrophils and macrophages in the mucosa, along with haemorrhagic serosa were evident on the 3rd day of ulceration. Treatment with BT, TF, and Omez for 3 days reduced the number of inflammatory cells and mucosal congestion, and increased the number of healthy normal cells in the gastric mucosa, submucosa, serosa, and muscle layers. Mucosal hyperplasia along with cryptic proliferation with no frank denudation was the major hallmark of the treatment. The effect of TF was slightly better amongst the test samples. The histological photographs of stomach sections of the 3rd day-groups of normal, ulcerated, and treated mice are shown in Figure 2.

### 3.3. BT and TF Alters Various Gastrointestinal Biochemical Parameters

#### 3.3.1. BT and TF Reduce the Oxidative Stress, Caused by Gastric Ulceration

**Figure 3:**
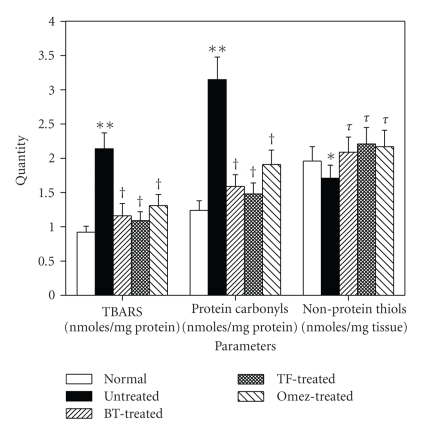
The effect of BT, TF, and Omez on the levels of TBARS, protein carbonyls, and nonprotein thiol (NP-TSH) in the ulcerated gastric tissues of mice. Ulceration in the mice was induced by indomethacin (18 mg/kg, *p. o*.). Treatment was carried out for 3 days with BT (40 mg/kg), TF (1 mg/kg), and Omez (3.0 mg/kg) and the parameters were determined by spectrophotometry. The values are mean ± S.E.M. of three independent experiments, each with 5 mice per group. *P < 0.05, **P < 0.001, compared to normal group; ^*τ*^ *P* < 0.05, ^†^ *P* < 0.01, compared to ulcerated group.

Indomethacin administration markedly stimulated lipid peroxidation in gastric tissues, and the TBARS content was elevated by 132.6% on 3rd day, compared to the normal value. BT and TF reduced it by 45.8% and 49.1%, respectively, compared to the group II mice. The effect of Omez (38.8%) was less than that of BT and TF. Compared to the normal value, the protein carbonyl content of the ulcerated mice was increased (154%) on the 3rd day of ulceration. BT and TF reduced it by 49.5% and 53%, respectively, while Omez reduced it by 39.4%, compared to the group II mice. Likewise, ulceration decreased (12.8%) NP-TSH, compared to the normal value. All the test samples increased it significantly, compared to that of the ulcerated mice. The results are presented in Figure 3.

**Figure 4:**
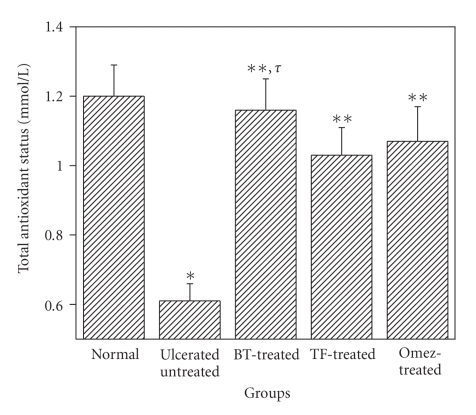
Depletion of the total antioxidant status (TAS) in mice plasma due to indomethacin-induced acute gastric ulceration, and its prevention by BT, TF, and Omez. Ulceration in the mice was induced by indomethacin (18 mg/kg, *p. o*.). Treatment was carried out for 3 days with BT (40 mg/kg), TF (1 mg/kg), and Omez (3.0 mg/kg) and TAS was determined by spectrophotometry. The values are mean ± S.E.M. of three independent experiments, each with 5 mice per group. *P < 0.01, compared to normal group; **P < 0.001, compared to ulcerated group; ^*τ*^ *P* < 0.05, compared to Omez-treatment.

The plasma TAS level in the group II mice was significantly less 49.6%, compared to the normal value (Figure 4). However, treatment with BT, TF, and Omez for 3 days augmented it by 90.2%, 68.9%, and 75.4%, respectively, compared to that of the untreated mice. The result with the TF treatment was significantly different from the other treatments.

#### 3.3.2. BT and TF Augment the Depleted Gastric Mucin Due to Ulceration

**Figure 5:**
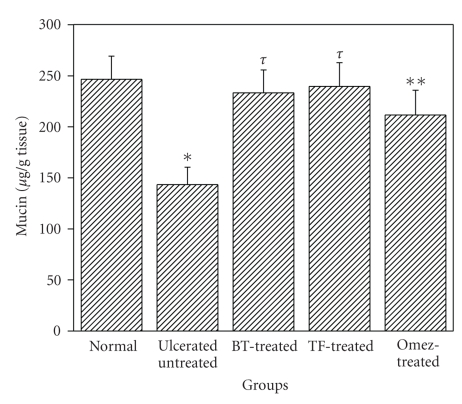
Depletion of mucin level in mice stomachs due to indomethacin-induced acute gastric ulceration and its prevention by BT, TF, and Omez. Ulceration in the mice was induced by indomethacin (18 mg/kg, *p. o*.). Treatment was carried out for 3 days with BT (40 mg/kg), TF (1 mg/kg), and Omez (3.0 mg/kg) and the gastric mucin was determined by assaying the tissue bound alcian blue. The values are mean ± S.E.M. of three independent experiments, each with 5 mice per group. *P < 0.01, compared to normal group; **P < 0.05, ^*τ*^ *P* < 0.01, compared to ulcerated group.

Compared to the normal level, ulceration reduced the mucin level by 41.9%. Treatment with BT and TF restored it to normalcy, while the effect of Omez was marginally less (Figure 5). 

#### 3.3.3. BT and TF Increase PGE Synthesis by Augmenting the Expressions of COX Enzymes

**Figure 6:**
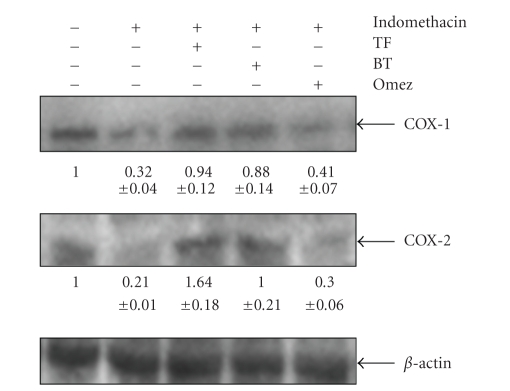
Immunoblots of the COX-1 and COX-2 expressions in the stomach tissues of normal, ulcerated, and treated mice. Ulceration in the mice was induced by indomethacin (18 mg/kg, *p. o*.). Treatment was carried out for 3 days with BT (40 mg/kg), TF (1 mg/kg), and Omez (3.0 mg/kg). The bands were quantified with respect to that of *β*-actin bands, using a Kodak Gelquant software. The values (arbitrary unit, mean ± S.E.M.) are the density scanning results of three independent experiments, considering that of normal mice as 1.

The western blots of COX-1 and COX-2 expressions in the gastric mucosa of the normal, ulcerated and drug (BT-, TF- or Omez-) treated mice are shown in Figure 6. The blot of normal gastric tissues showed very strong COX-1 expression with a low intensity band for COX-2. Ulceration depleted (*P* < 0.001) the expressions of gastric COX-1 and COX-2 by 68% and 79%, respectively, compared to that in normal mice. Treatment with BT and TF increased (*P* < 0.001) both COX-1 (3 fold) and COX-2 (8 fold) almost equally, compared to the untreated group. In contrast, the effect of Omez was much less, increasing the expressions of COX-1 (28.1%, *P* < 0.05) and COX-2 (80.97%, *P* < 0.001), compared to the untreated group. The effects of BT and TF were significantly different from that of Omez.

**Figure 7:**
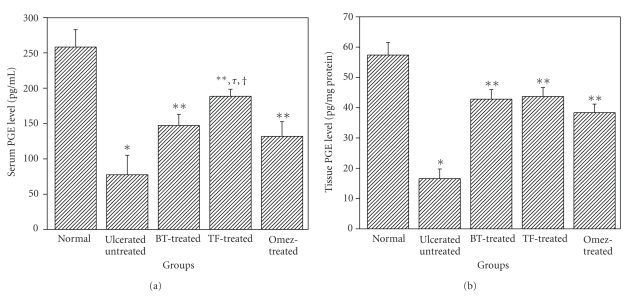
Comparative ability of BT, TF, and Omez in regulating the PGE synthesis in acute gastric ulcerated mice. The PGE levels were measured using ELISA. (a) The serum PGE level. (b) The tissue PGE level. The values are mean ± S.E.M. of three independent experiments, each with 5 mice per group. *P < 0.001, compared to normal group; **P < 0.001, compared to ulcerated group; ^*τ*^ *P* < 0.01, compared to Omez-treatment; ^†^ *P* < 0.05, compared to BT-treatment.

The serum PGE level was decreased (70%) on the 3rd day of ulceration, compared to that in normal mice (Figure 7). Treatment with BT, TF, and Omez increased it by 89.8%, 143.2%, and 70.2%, respectively, the effect of TF being significantly better than that of Omez and BT. Ulceration also reduced (71%) the mucosal PGE, compared to that in normal mice. Treatment with BT, TF, and Omez increased the mucosal PGE by 157.7%, 163.1%, and 131.1%, respectively, compared to that in the ulcerated mice.

## 4. Discussion

The concept of gastric ulcer management is changing fast. Besides uncontrolled acid secretion, damage to the mucosal defense is also believed to be responsible for the disease. Understanding the role of factors, contributing to the mucosal defense might lead to the designing of new antiulcer drugs. Therefore, we evaluated the healing efficacy of the common dietary factor, BT and its major constituent, TF against the indomethacin-mediated gastric ulceration in mice, and assessed their role in augmenting the mucosal defense.

Our macroscopic and histopathological results showed marked gastric mucosal damage in mice, on the 3rd day after indomethacin administration. This led to elongated haemorrhagic lesions, confined to the glandular portion, with highest subjective ulcer-scoring. The partial natural healing observed in the untreated control mice revealed that the ulceration was acute. However, the natural healing was much slower compared to that observed in the mice treated with the test samples. Both BT and TF showed impressive mucosal healing, TF being more potent than BT. Under an optimized three-day treatment regime, BT (40 mg/kg), TF (1 mg/kg), and Omez (3 mg/kg) produced similar ulcer healing. 

Amongst various factors, oxidative stress (OS) has been implicated for the induction and pathogenesis of the indomethacin-mediated gastroduodenal injury [36, 37]. Extensive research has proved that antioxidants might be effective not only in protecting against gastric mucosal injury, but also inhibiting progression of gastric ulcer. Our results showed increased accumulation of TBARS and protein carbonyls along with depletion of NP-TSH in the gastric tissues, after the indomethacin administration. These were consistent with the earlier reports on the indomethacin-induced gastropathy [38, 39]. The induced lipid peroxidation might cause increased glutathione consumption. The sulphydryl compounds help in recycling endogenous antioxidant vitamins, thereby, preventing lipid peroxidation. More importantly, they also protect mucus by preventing rupture of the disulfide bridges that join the mucus subunits and maintain its structural integrity. The decrease in endogenous thiol (glutathione) in ethanol-induced gastric injury and its role in mucosal protection has been demonstrated earlier [39]. Both BT and TF provided nearly similar and significant suppression of the oxidative damages to the biomacromolecules, compared to that observed in natural recovery. This might decrease the ulcer progression and promote healing of gastric lesions induced by acute intake of indomethacin.

Compared to the individual oxidative markers, assay of the plasma TAS level provides a better index of the body's total systemic antioxidant defense comprising of the enzymes, such as superoxide dismutase and the selenium-containing glutathione peroxidase as well as nonenzymic antioxidants (radical scavengers and chelating agents), and their synergistic interaction [40]. Our results on the reduced plasma TAS level of the indomethacin-administered mice revealed severe oxidative stress. The test samples improved the parameter markedly, TF being significantly more potent than BT and Omez. 

Depletion of gastric mucosal mucin level also contributes to the NSAID-mediated gastropathy. Maintenance of mucus production may provide partial but significant protection against reactive oxygen metabolites. Our results revealed that stomach ulceration reduced the gastric mucin content. This might reduce the ability of the mucosal membrane to protect the mucosa from physical damage and back diffusion of hydrogen ions [41], and hinder epithelial recovery. Treatment with BT, TF, and Omez significantly accelerated ulcer healing, which was associated with an increase in the mucin content of the gastric mucosa. Amongst the test samples, BT and TF, but not Omez, restored the mucin level to normalcy.

**Figure 8:**
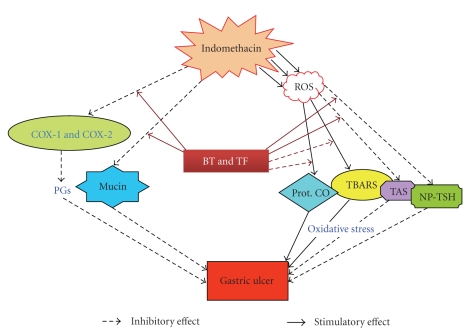
Schematic representation of the mechanism of the ulcer-healing action of BT and TF.

The NSAIDs exert both their therapeutic and toxic effects mainly by decreasing the levels of circulating PGE at the gastric mucosa via inhibition of the COX isozymes. The reduced level of PGs is known to cause gastric ulceration and also exacerbate preexisting gastric ulcers in rodents and humans [3, 42]. PGs stimulate mucus and bicarbonate secretion as well as mucosal blood flow, and induce angiogenesis [43]. All these factors contribute to accelerated ulcer healing. Our immunoblots revealed reduced expressions of COX-1 and COX-2 at peak ulceration, associated with reduced synthesis of serum and mucosal PGE. Treatment with BT and TF increased the expressions of both the enzymes, the effect being more predominant on COX-2. Omez showed less effect on these enzymes. The test samples augmented the serum and mucosal PGE that correlated well with their respective abilities to regulate the expressions of the COX isoforms. The enhanced PG synthesis by BT and TF might stimulate the EP4 receptor-mediated mucin synthesis [44] and inhibit the neutrophil-mediated free radicals generation [45]. In separate experiments, we did not observe any antisecretory property of BT and TF. The mechanism of the healing action of BT and TF against the indomethacin-mediated gastric ulceration can be summarized as shown in Figure 8.

The tea decoction is a complex mixture of products comprising of a group of biopolymers, theaflavins, and the water-soluble thearubigins [27]. Hence, we did not attempt to analyze the tea decoctions completely. However, our HPTLC and HPLC analyses revealed caffeine (70%), theaflavins (18%), and catechins (10%) as the major components. Of these, caffeine is suggested to aggravate an existing ulcer by stimulating acid secretion. However, the stimulation of stomach acid cannot be attributed solely to caffeine [46]. Earlier, the tea catechins have been reported to prevent and heal gastric ulcers caused by several ulcerogens [18, 19]. However, our results clearly demonstrated that TF could account for almost the entire healing activity of BT. The catechins might have a synergistic effect in the healing action of BT against indomethacin-induced gastric ulceration in mice. The other water soluble compounds of BT including the thearubigins of undefined chemical structures were not included for the studies, since these were envisaged to be too polar for membrane penetration.

## 5. Conclusions

Overall, despite the purported gastrotoxicity of BT, our results clearly revealed its healing ability against indomethacin-induced stomach ulceration and established TF as the active principle. The results are consistent with the anti-inflammatory property of TF [47]. Based on the available evidence, the therapeutic effect of these test samples could be related to their antioxidant, mucin-protecting, and PGE-enhancing properties.

## Figures and Tables

**Table 1:** Dose and day-dependent healing capacities of BT and TF against indomethacin-induced stomach ulceration in mice^*a*^.

Group	Drug dose (mg/kg)	Period of treatment (days)	Macroscopic damage scores (MDS)^b^	MDS reduction (%)^c^
3rd day-ulcerated untreated	—		3.39 ± 0.24	0
5th day-ulcerated untreated			1.21 ± 0.15	
7th day-ulcerated untreated			0.68 ± 0.07	
BT-treated	10	3	2.64 ± 0.22*	22.13
BT-treated	20	3	1.89 ± 0.17**	44.25
BT-treated	30	3	1.47 ± 0.15^†^	56.64
BT-treated	40	3	0.88 ± 0.06^†,#^	74.05
BT-treated	50	3	0.83 ± 0.07^†,#^	75.52
BT-treated	40	7	0.55 ± 0.12	83.78
TF-treated	0.5	3	1.12 ± 0.13^†^	66.96
TF-treated	1	3	0.81 ± 0.07^†,Ψ^	76.4
TF-treated	2.5	3	0.74 ± 0.06^†,Ψ^	78.17
TF-treated	4	3	0.62 ± 0.07^†,Ψ^	82.3
TF-treated	5	3	0.53 ± 0.06^†,Ψ^	84.36
TF-treated	1	7	0.46 ± 0.08	86.44

^a^Stomach ulceration in mice was induced by oral administration of indomethacin (18 mg/kg). Different doses of BT and TF were used for these experiments. ^b^The MDS were measured on the 3rd, 5th, and 7th day after indomethacin administration and the values are mean ± S.E.M of three independent experiments, each with 5 mice/group. ^c^Considering a MDS value of 100 for the 3rd day untreated mice. *P < 0.05, **P < 0.01, ^†^ *P* < 0.001 compared to ulcerated mice; ^#^ *P* < 0.05 compared to BT (30 mg/kg) treatment; ^Ψ^ *P* < 0.05 compared to TF (0.5 mg/kg) treatment.
